# Pain Mystery Score Beliefs: A Comparison of Fibromyalgia and Rheumatoid Arthritis

**DOI:** 10.1155/2014/593507

**Published:** 2014-12-09

**Authors:** Robert Ferrari, Anthony Science Russell

**Affiliations:** Department of Medicine, University of Alberta, 13-103 Clinical Sciences Building, Edmonton, AB, Canada T6G 2P4

## Abstract

*Objectives*. To compare the mysteriousness scores of the Pain Beliefs and Perceptions Inventory in fibromyalgia.* Methods*. Two cohorts of patients, one with fibromyalgia (FM) and one with rheumatoid arthritis (RA), completed the Mystery Scale component of the Pain Beliefs and Perceptions Inventory to determine whether subjects in the two diagnostic groups had significantly different scores on the Mystery Scale.* Results*. A total of 126 subjects (64 FM, 62 RA) completed all questionnaires. The FM group had a greater percentage of female subjects, more severe pain, more severe anxiety, more severe depression, and a higher perceived injustice score. When the RA and FM group scores for the Mystery Scale were adjusted for age, sex, pain severity, HADS scores, and perceived injustice scores, the FM group still had a higher Mystery Scale score.* Discussion*. Fibromyalgia is associated with a higher level of perception of mysteriousness in the Pain Beliefs and Perceptions Inventory than is seen with rheumatoid arthritis. This difference appears to be independent of levels of pain, depression, anxiety, and perceived injustice. This sense of mysteriousness may reflect a lack of understanding of pain in fibromyalgia as previously reported and may be an area to be addressed in therapy.

## 1. Introduction

It has previously been shown that fibromyalgia patients differ from other widespread pain patients in that fibromyalgia patients are more likely to perceive a much greater degree of difficulty both in understanding the cause of their pain and in explaining the cause of their pain to others [1]. This was concluded after utilization of the Understand Pain Scale and the Explain Pain Scale. That is, subjects were asked to indicate on a Likert scale the degree to which they “understand the cause of their pain (the reason they have pain)” (the Understand Pain Scale) and the degree to which they “can explain the cause of their pain (the reason they have pain) to others” (the Explain Pain Scale). Using these scales and controlling for age, gender, and duration of pain, fibromyalgia subjects had higher scores than comparator groups, which included whiplash-associated disorder, tendinitis/bursitis, and osteoarthritis. Although a handful of rheumatoid arthritis subjects were included in this study [1], they were few, and to date there is no further validation of the Understand Pain Scale and Explain Pain Scale, in patients with painful disorders.

On the other hand, the Pain Beliefs and Perceptions Inventory has had a much more detailed study of its construct [2, 3]. Specific items of this inventory comprise the mysteriousness construct, asking subjects specifically about the extent to which they agree with the statements: “No one's been able to tell me exactly why I'm in pain,” “My pain is confusing to me,” “I do not know enough about my pain,” and “I cannot figure out why I'm in pain.” As depression, anxiety, and perceived injustice have high correlations with pain in both fibromyalgia and rheumatoid arthritis patients [4], a comparison of the mysteriousness scores in these two groups necessitates controlling for these confounders.

Rheumatoid arthritis is useful as a comparator group for fibromyalgia cohorts [1, 5–7], in part because rheumatoid arthritis sufferers share many of the same symptoms, especially widespread pain and fatigue. At the same time, rheumatoid arthritis is a far less controversial condition. That is, fibromyalgia has a controversial nature that presents a challenge for both patients and physicians [8–11]. It is possible that this issue and the controversial nature of fibromyalgia contribute to this sense of mysteriousness or lack of understanding expressed by sufferers. The explanations for fibromyalgia have varied as polar opposites. Some researchers have invoked explanations such as central sensitization or viral syndrome [12, 13], while others regard fibromyalgia as a psychocultural illness [14]. While there are controversies in rheumatoid arthritis, this extreme spectrum of polar views does not exist. This polarity may in part help to explain the difficulties that fibromyalgia patients have in understanding or explaining their suffering to others [1]. Fibromyalgia patients have remarked that the fact that the medical community cannot agree on the nature of fibromyalgia and cannot resolve the controversies makes it very difficult for fibromyalgia patients to know what to believe about their illness [15].

The purpose of this study was therefore to quantify the degree to which fibromyalgia patients perceive mysteriousness in the cause of their pain and to compare this to the perceptions of rheumatoid arthritis patients.

## 2. Materials and Methods

### 2.1. Subjects

The subjects are those described in a previous study that has been part of a larger effort to determine the clinical utility of various questionnaires in the care of fibromyalgia patients [4]. These subjects were recruited from two adult rheumatology practices in Edmonton, Alberta, Canada, serving a catchment area of 1.5 million population. The subject groups consist of consecutive patients presenting with a confirmed diagnosis of rheumatoid arthritis via the 1987 American College of Rheumatology Criteria [16] (RA group) and the other group with a diagnosis of fibromyalgia via the 2010 Modified American College of Rheumatology Criteria [17] (FM group).

### 2.2. Inclusion/Exclusion Criteria

The authors reviewed the charts of all consecutively seen subjects who had a confirmed diagnosis of either rheumatoid arthritis or fibromyalgia. Inclusion criteria also included age over 18. Exclusion criteria included the following: being unable to read English; having lower than grade 8 level education; having suspected or known malignancy, or other serious illness; and having both fibromyalgia and rheumatoid arthritis.

### 2.3. Data Collection

From January 2013, the authors had been, as part of clinical care, routinely asking chronic pain patients, including rheumatoid arthritis and fibromyalgia patients to complete various instruments in an effort to determine their utility in practice. As part of a practice audit, the charts of rheumatoid arthritis and fibromyalgia patients were reviewed until May, and data gathered regarding age, gender, and assessment responses (see below). This data was anonymised for the purpose of further analysis. Thus, no data was then available on duration of condition, comorbidities, history of trauma, litigation or compensation status, psychiatric diagnoses, disability status, or treatment.

### 2.4. Instruments

#### 2.4.1. Numerical Pain Scale

Subjects were asked to specify, on average, how much pain they had because of their condition over the past week. They circled a number between 0 and 10, with 0 being labelled as “no pain” and 10 being labelled as “worst pain imaginable.”

#### 2.4.2. Injustice Experience Questionnaire

The Injustice Experiences Questionnaire (IEQ) was used, as described by Sullivan et al. [18], to measure injury-related perceptions of injustice. Subjects were asked to rate the frequency with which they experienced each of 12 pain-related thoughts on a 5-point scale, ranging from 0 (never) to 4 (all the time). Examples of items include the following: “Most people do not understand how severe my condition is,” “My life will never be the same,” “I am suffering because of someone else's negligence,” and “It all seems so unfair.” The maximum score (maximal perceived injustice) is 48 and the minimum is zero. The scale can be further divided and scored along two factors: blame/unfairness and severity/irreparability of loss. The questionnaire instructions were modified to include the phrase “injury/illness,” as the original questionnaire referred only to injury, which would not be appropriate in this sample.

#### 2.4.3. Hospital Anxiety and Depression Scale

The Hospital Anxiety and Depression Scale (HADS) was originally developed by Zigmond and Snaith [19] to determine the levels of anxiety and depression that a patient is experiencing. The HADS is a fourteen-item scale (seven of the items relate to anxiety and seven relate to depression). Subjects rate the severity of depression or anxiety symptoms on a scale of 0–3 (3 being most severe) and receive a score on each of the depression and anxiety scales of 0–21.

#### 2.4.4. Mysteriousness

This was assessed using items 1, 4, 8, and 14 from the Pain Beliefs and Perceptions Inventory [2]. These items comprise the mysteriousness construct of that inventory. We did not include the full Pain Beliefs and Perceptions Inventory so as not to overburden subjects in this pilot study. Instead, four statements from the Pain Beliefs and Perceptions Inventory were presented to each subject with a Likert scale ranging comprised of Strongly Disagree (−2), Disagree (−1), Agree (1), and Strongly Agree (2). The numbers in parentheses are totaled to arrive at a mysteriousness score. The total scores may thus range from −8 to +8. The more positive the total, the more mysteriousness the subject perceives in regard to their pain.

### 2.5. Data Analysis

Initially, all data records were reviewed in order to ascertain if any data issues, such as missing data, outliers, or out of range values, existed within the data set. Subjects with missing diagnosis or data were removed from analysis along with subjects diagnosed with both fibromyalgia and rheumatoid arthritis. After data cleaning, the subjects were divided into two groups (RA group and FM group) as the main variable. The mean age, gender distribution, and mean scores of the assessments were calculated for each group. Inferential statistics were then used to determine whether subjects in the two diagnostic groups had significantly different scores on the Mystery Scale items. This was done univariately using *t*-tests and after adjusting for potential confounders (sex, age, pain, HADS depression, HADS anxiety, and perceived injustice) using ANCOVA. Adjusted mean scores were determined by estimating marginal means for the diagnosis variable after entering potential confounders into the ANCOVA model. We also examined the adjusted association between diagnostic group and Mystery Scale scores using multivariable linear regression. Our final models were built in a blocked fashion by initially entering diagnostic category into the model and then entering other variables simultaneously using a stepwise strategy (*P*-to-enter ≤ 0.05; *P*-to-remove ≥ 0.10). Relevant regression assumptions were examined including normality, linearity, and independence. A 0.05 alpha level was used to judge statistical significance. All analyses were conducted using IBM SPSS 20 (Chicago, IL).

### 2.6. Sample Size Calculation

We have no previous studies on this topic to assess sample size. However, we had a fixed sample from our practice audit, which limited the sample size. Considering the number of subjects in the study sample previously reported to be 64 rheumatoid arthritis subjects and 62 fibromyalgia subjects, we have calculated that, given a standard deviation in Mystery Scale scores of 5.5 (observed for the two groups combined), we had an 80% power to detect an absolute difference of 2.8 in the total Mystery Scale scores between groups.

## 3. Results

### 3.1. Data Cleaning

Initially a total of 147 charts were available for review. Of these, 9 subjects were excluded (6 because of lack of English language skills, 1 because of malignancy, and 3 because of having both RA and FM). This left 138 subjects for analysis. Of these, an additional 12 were excluded from analysis because of significant missing data. Thus, a total of 126 subjects (64 RA and 62 FM) had complete data available for analysis.

### 3.2. Descriptive Statistics

The gender distribution, mean age, and unadjusted assessment scores are shown in Table 1. The FM group had a greater percentage of female subjects, more severe pain, more severe anxiety, more severe depression, and a higher perception of injustice. In unadjusted analysis, as shown in Table 1, the FM group also had a higher mean Mystery Scale scores. That is, the unadjusted mean for the FM group Mystery Scale score was 2.9, indicating that they were more likely to agree that their pain was not understood or had greater mysteriousness. In contrast, the RA group had an unadjusted mean Mystery Scale score of −4.5. This indicates that they tended to disagree with the notion of having mysteriousness to their pain.

### 3.3. Regression Modelling

After adjustments by sequentially adding the confounders of age and sex, pain, HADS depression, HADS anxiety, and perceived injustice, a significant difference remains between groups. That is, the adjusted mean for the FM group Mystery Scale score was 1.6 and the RA group had an unadjusted mean Mystery Scale score of −3.4. This indicates again that the FM group supported the notion of having a mysteriousness concerning their pain when compared to the RA group.

## 4. Discussion

This study of a referred cohort of subjects with either fibromyalgia or rheumatoid arthritis shows that fibromyalgia patients are much more likely than rheumatoid arthritis patients to endorse the belief that they have a degree of mysteriousness concerning their pain. This remains the case even after adjusting for confounders that included severity of pain, for example. The results of this study are in agreement with a previous study [1] using novel Understand Pain and Explain Pain Scales, suggesting that understanding or explaining pain is perceived to be more difficult in fibromyalgia sufferers.

A narrative approach to understanding fibromyalgia patients affirms that both the wide array of symptoms in fibromyalgia and the variability in the illness course make it an illness difficult for patients themselves to understand and even more difficult to explain to others [10, 11]. Fibromyalgia patients have long been described as having a disorder that lacks face-validity [20]. The cause of their illness remains controversial despite an extensive number of therapies and considerable progress in pain research, specifically as it pertains to fibromyalgia [6, 21–27]. The feeling or implication that one's symptoms, especially pain, are described as “medically unexplained” is of concern to patients, to the point of being offensive [7]. Fibromyalgia patients report a wide array of symptoms, frequent fluctuation in the presence and severity of symptoms, and unpredictable exacerbations [28–31].

Labelling the condition fibromyalgia, in itself, does not necessarily convey greater meaning or understanding for this suffering. Patients have indicated exactly that while the diagnosis may confer some legitimacy, it does not improve their understanding of their own illness, nor help them explain their illness to others [32, 33], nor does the label appear to assist or comfort physicians [34]. What the current study confirms is that this mysteriousness or lack of understanding around fibromyalgia persists even if one controls for other factors, like pain severity, anxiety, depression, and a sense of injustice.

There are a number of limitations to this study. First, the psychometrics of the Mystery Scale score used without the remainder of the Pain Beliefs and Perceptions Inventory are not known. Further study may be required to ascertain the reproducibility, reliability, and validity of using selected items from this inventory in various settings, adding the other constructs of this inventory as confounders in the analysis. The questionnaires we used, however, were part of a practice audit and assessment. They were thus used in a way that, through brevity, would be most applicable in the clinical setting.

In terms of selection bias, these subjects were from two clinics. There may be a selection bias in this group that affected their sense of mysteriousness because they were indeed a referral sample, possibly with more severe pain, or seeking more explanation. A primary care sampling of fibromyalgia subjects may have yielded different results. We did not track the scores used to confirm the FM diagnosis via the Modified 2010 ACR criteria, which may have told us how severe these patients were in terms of polysymptomatic distress. Still, we did adjust for illness severity to an extent, in that we adjusted for pain, depression, and anxiety. In addition, we did not measure cognitive dysfunction in the subjects. This could explain why fibromyalgia patients have difficulty understanding their pain, but narrative and other qualitative studies of fibromyalgia patients clearly indicate that fibromyalgia patients are very much capable of logically and earnestly processing their illness and suffering: despite this, they find their illness often inexplicable [10, 11]. Cognitive dysfunction is frequently reported in other widespread pain subjects who do not have fibromyalgia, as well [35]; yet, this does not prevent them from holding the belief that they understand the cause of their pain and can readily explain it to others [1].

The question remains as to what to do with the knowledge that fibromyalgia patients perceive a greater perception of mysteriousness to their pain. To the extent that this mysteriousness might contribute to their distress, future studies could examine whether encouraging patients not to seek explanations (but rather solutions) for their pain may help their condition. As far off as such studies may be, the Mystery Scale may be used to inform both clinical practice and research efforts. It may also be that there is no solution to this problem because the research literature is the problem. The mysteriousness of pain perceived by fibromyalgia patients may simply be a reasonable reflection of the polarity reported by researchers holding divergent views on the topic. Fibromyalgia sufferers may be the unfortunate victims of this tug-of-war. To that extent, there may be no mystery at all.

## Figures and Tables

**Table 1:** Patient characteristics according to diagnostic group.

	Entire sample	Rheumatoid arthritis	Fibromyalgia
	(*n* = 126)	(*n* = 64)	(*n* = 62)
	Mean (std dev.) or percent		
Sex (% female)	72.2	54.7	90.3
Age (years)	48.9 (12.8)	53.7 (12.9)	43.9 (10.7)
Pain Visual Analogue Scale (out of 10)	5.8 (2.7)	4.3 (2.7)	7.3 (1.5)
Injustice Experiences Questionnaire total	17.9 (10.3)	14.4 (9.8)	21.6 (9.7)
HADS—anxiety (*n* = 126)	7.5 (4.6)	5.8 (4.2)	9.2 (4.3)
HADS—depression (*n* = 124)	8.5 (3.5)	7.3 (3.5)	9.7 (3.1)
Mystery Scale	−0.8 (5.5)	−4.5 (3.9)	2.9 (4.4)

All descriptive variables demonstrated statistically significant differences between groups.
